# Hepatocytic Ballooning in Non-alcoholic Steatohepatitis: Bridging the Knowledge Gap and Charting Future Avenues

**DOI:** 10.7759/cureus.45884

**Published:** 2023-09-25

**Authors:** Tanvi Singla, Komal N Muneshwar, Aniket G Pathade, Seema Yelne

**Affiliations:** 1 Community Medicine, Jawaharlal Nehru Medical College, Datta Meghe Institute of Higher Education and Research, Wardha, IND; 2 Research and Development, Jawaharlal Nehru Medical College, Datta Meghe Institute of Higher Education and Research, Wardha, IND; 3 Nursing, Shalinitai Meghe College of Nursing, Datta Meghe Institute of Higher Education and Research, Wardha, IND

**Keywords:** personalized medicine, therapeutic targets, diagnosis, molecular pathways, fibrosis, inflammation, nash progression, nafld, hepatocytic ballooning, non-alcoholic steatohepatitis

## Abstract

Non-alcoholic steatohepatitis (NASH) is emerging as a significant global health concern, characterized by hepatic lipid accumulation, inflammation, and hepatocellular injury. Hepatocytic ballooning, a histological feature of NASH, has gained prominence for its role in disease progression and potential as a therapeutic target. This review provides an overview of the current knowledge regarding hepatocytic ballooning in NASH, highlighting the key molecular and cellular mechanisms implicated in its development. We delve into the intricate interplay of metabolic dysregulation, oxidative stress, and lipid toxicity as drivers of hepatocytic ballooning, shedding light on the pathways responsible for its initiation and perpetuation. Furthermore, we explore the diagnostic challenges associated with hepatocytic ballooning and its significance as a prognostic indicator in NASH patients. While hepatocytic ballooning holds promise as a therapeutic target, this abstract discusses the various experimental and clinical approaches to ameliorate this histological hallmark. Potential interventions, including lifestyle modifications, pharmacological agents, and emerging therapies, are evaluated in terms of their efficacy and safety profiles. In conclusion, this review underscores the need to bridge the knowledge gap surrounding hepatocytic ballooning in NASH and emphasizes its importance in understanding disease pathogenesis and progression. By charting future research avenues and clinical strategies, we aspire to advance our comprehension of NASH and ultimately improve patient outcomes in this rapidly evolving field of hepatology.

## Introduction and background

Non-alcoholic fatty liver disease (NAFLD) is one of the most prevalent chronic hepatic conditions globally, mirroring the alarming surge in obesity and metabolic syndrome cases. NAFLD encompasses a spectrum of hepatic disorders, ranging from the relatively benign simple steatosis, characterized by excessive lipid accumulation within hepatocytes, to the more severe non-alcoholic steatohepatitis (NASH). NASH is distinguished by inflammation, hepatocellular injury, and the potential progression to fibrosis [1,2].

NAFLD has become a significant public health concern, afflicting an estimated one-quarter of the global populace. This disease spectrum spans from isolated hepatic steatosis, often perceived as less menacing, to the more aggressive NASH, which can potentially evolve into cirrhosis and hepatocellular carcinoma. Differentiating between these conditions is paramount, as it governs clinical management strategies and prognostic outcomes [3].

Among the manifold histological features characterizing NASH, hepatocytic ballooning has assumed a central and distinctive role. Hepatocytic ballooning, characterized by the swelling and morphological alterations of hepatocytes, signifies a state of cellular stress and dysfunction. While ballooning is a hallmark of NASH, the precise underlying mechanisms and implications continue to be subjects of ongoing investigation [4,5].

Hepatocytic ballooning transcends its mere aesthetic appearance; rather, it represents a pivotal stage in the pathogenesis of NASH. Ballooned hepatocytes denote cellular injury and stress responses, often foreshadowing apoptotic cell demise and ensuing inflammation. Unraveling the triggers and consequences of hepatocytic ballooning holds pivotal significance in comprehending the trajectory of NASH progression and formulating targeted therapeutic interventions [6,7].

The intricate nature of hepatocytic ballooning has engendered multiple knowledge gaps in our comprehension of NASH development. This review endeavors to consolidate the existing body of literature on hepatocytic ballooning within the context of NASH and its implications. By undertaking a critical appraisal of the current state of knowledge, we aim to pinpoint areas necessitating further investigation and propose potential avenues for exploration. This review strives to bridge the lacunae in our understanding of hepatocytic ballooning and its role in NASH, ultimately contributing to advancing clinical management and therapeutic strategies.

## Review

Pathophysiology of NASH

NAFLD Pathogenesis and Risk Factors

NAFLD is a multifaceted condition with a pathogenesis influenced by genetic, environmental, and metabolic factors. The core of NAFLD's intricate development is the accumulation of lipids, predominantly triglycerides, within liver cells, resulting from a delicate disruption in the balance between lipid uptake, de novo synthesis, and export from the liver [8,9].

Genetic predisposition significantly contributes to an individual's susceptibility to NAFLD. Variations in genes related to lipid metabolism, insulin signaling, and inflammation can affect how the liver manages lipids and responds to metabolic challenges. These genetic factors can increase the likelihood of lipid accumulation and inflammation, exacerbating NAFLD development [10,11].

Environmental factors are also pivotal in initiating and advancing NAFLD. Modern sedentary lifestyles and high-caloric diets lead to excessive calorie consumption, especially from fats and sugars. This surplus of calories and reduced physical activity disrupts the balance between energy intake and expenditure. The excess energy is stored as triglycerides within liver cells, initiating the series of events characterizing NAFLD's pathogenesis [12,13].

Furthermore, the interplay between genetic susceptibility and environmental factors complicates NAFLD development. Individuals with specific genetic variants may be more prone to lipid accumulation when exposed to a high-caloric diet or a sedentary lifestyle. Similarly, genetic factors can influence how an individual responds to metabolic stressors like insulin resistance, further impacting the progression of NAFLD [14].

Distinctive Features of NASH Including Inflammation, Hepatocellular Injury, and Fibrosis

NASH represents a complex and dynamic progression from the initial phase of simple steatosis to a more aggressive phenotype marked by various distinctive pathological changes. A key hallmark of NASH is hepatocellular injury, a consequence of cellular stress responses triggered by excessive lipid accumulation. This injury sets the stage for a series of events, including oxidative stress, inflammation, and disruptions in metabolic pathways [15,16].

The inflammatory aspect of NASH is characterized by the infiltration of immune cells into the liver, giving rise to localized inflammation within the hepatic lobules. This lobular inflammation contributes to the unique histological pattern observed in NASH biopsies, often featuring clusters of inflammatory cells surrounding ballooned hepatocytes. These inflamed regions can further exacerbate hepatocellular injury and progress the disease [17,18].

Hepatocellular ballooning, a prominent hallmark of NASH, entails the swelling and distortion of hepatocytes due to various insults, including oxidative stress and metabolic dysfunction. These ballooned hepatocytes exhibit altered morphology, increased cytoplasmic volume, and pale staining on histopathological examination. Ballooning signifies a state of cellular stress and impending damage, frequently serving as a precursor to apoptosis and heightened inflammation. This ballooning phenomenon is a pivotal point in the progression of NASH, indicating an increased risk of more severe outcomes [19].

As the disease advances, fibrosis - the excessive accumulation of extracellular matrix - becomes increasingly prominent. Fibrosis develops in response to ongoing tissue injury and inflammation, activating stellate cells and leading to collagen deposition. The extent of fibrosis varies among individuals and can progress to advanced stages, ultimately culminating in cirrhosis. Cirrhosis is characterized by extensive fibrotic scarring and substantial disruption of liver architecture [20].

Role of Oxidative Stress, Lipotoxicity, and Insulin Resistance in NASH Development

The complex interplay between oxidative stress, lipotoxicity, and insulin resistance intricately drives NASH development and progression. Oxidative stress, a consequence of the imbalance between the generation of reactive oxygen species (ROS) and the body's antioxidant defense mechanisms, plays a pivotal role in NASH pathogenesis. In the context of NASH, the excessive accumulation of lipids within hepatocytes assumes a dual role in intensifying oxidative stress. Lipids serve as substrates for ROS production and disrupt normal mitochondrial function, creating an environment conducive to cellular damage [21].

Lipotoxicity, often defined as the adverse effects resulting from the excessive exposure of hepatocytes to lipids, further amplifies the cascade of events contributing to NASH. The accumulation of lipids surpasses the liver's physiological capacity to manage them appropriately, causing cellular signaling and metabolism disruptions. These disruptions, in turn, trigger inflammatory responses and apoptotic pathways, fostering the proinflammatory environment characteristic of NASH. Furthermore, lipotoxicity intertwines with oxidative stress, exacerbating mitochondrial dysfunction and cellular stress [22].

Insulin resistance, a hallmark of metabolic syndrome, exacerbates the detrimental effects of lipotoxicity on NASH development. Impaired insulin signaling compromises the liver's ability to regulate glucose and lipids effectively, leading to dysregulated lipid homeostasis. Consequently, elevated circulating levels of free fatty acids are deposited within hepatocytes, further fueling lipotoxicity and oxidative stress. This intricate interplay creates a vicious cycle in which insulin resistance promotes lipid accumulation, contributing to oxidative stress and inflammation [23].

Connection Between Hepatic Ballooning, Inflammation, and Disease Progression

Hepatocytic ballooning, a central histological hallmark within NASH, unveils a complex interplay between inflammation and the progression of this disease. These ballooned hepatocytes, distinctive in their altered morphology and cytoplasmic structure, display an increased susceptibility to apoptosis and a tendency to attract immune cell infiltration. In addition, these ballooned hepatocytes release various proinflammatory molecules, thereby intensifying the inflammatory environment inherent to NASH [24].

The link between hepatocytic ballooning and inflammation is further underscored by the influence of cytokines, particularly interleukin-1β (IL-1β) and tumor necrosis factor-α (TNF-α). These inflammatory mediators are elevated in NASH and play a pivotal role in initiating and perpetuating the ballooning phenotype. They contribute to the compromised cellular health observed in ballooned hepatocytes, exacerbating cellular stress responses. Consequently, hepatocytic ballooning serves as a nexus, bridging the molecular pathways of inflammation with the altered cellular state, ultimately contributing to the progression of NASH [25,26].

Furthermore, hepatocytic ballooning is proposed to represent a critical juncture in the continuum of NASH. Its severity has been demonstrated to correlate with the extent of inflammation, advancement of fibrosis, and clinical outcomes. As hepatocytic ballooning worsens, the delicate balance between cell survival and apoptosis becomes disrupted, tilting toward heightened cellular injury and subsequent inflammation. This concept of a tipping point underscores the clinical significance of hepatocytic ballooning as an indicator of disease severity and progression [27].

Hepatocytic ballooning: definition, assessment, and clinical implications

Hepatocytic ballooning, a prominent histological feature in NASH, is characterized by distinct morphological changes within hepatocytes. When observed under histological examination, hepatocytic ballooning is recognized by noticeable alterations in the appearance and structure of these liver cells. Ballooned hepatocytes typically exhibit specific features, including a rounded cellular shape, a noticeable increase in cytoplasmic volume, and a discernible change in cytoplasmic staining properties, often appearing pale and eosinophilic [16].

Although the exact molecular mechanisms underlying hepatocytic ballooning are not yet fully understood, researchers have a consensus that this phenomenon signifies a state of heightened cellular stress and disruption in the normal hepatocyte architecture. This stress response is often associated with disturbances in the integrity of the cytoskeletal framework, which provides structural support to cells. Additionally, dysfunction in cellular organelles such as mitochondria and endoplasmic reticulum (ER) is believed to contribute to the ballooning phenotype. These intracellular disturbances collectively demonstrate that hepatocytic ballooning extends beyond mere morphological changes and serves as a visible representation of the complex cellular processes involved in the development of NASH [28].

Evaluation Methods for Hepatocytic Ballooning (Histopathology, Imaging, and Biomarkers)

Histopathological assessment stands as the cornerstone for identifying and grading hepatocytic ballooning, representing the gold standard for clinical diagnosis. Hematoxylin and eosin (H&E) staining, in particular, provide pathologists with the means to visualize and quantify the characteristic morphological changes associated with ballooning. These changes include the distinct cytoplasmic swelling and the pale eosinophilic appearance of affected hepatocytes. Furthermore, specialized staining techniques like Periodic Acid-Schiff (PAS) staining can offer additional insights into glycogen accumulation, aiding in the differentiation of ballooning from other cellular alterations [29].

Recent advancements in imaging technologies have introduced non-invasive modalities for assessing hepatic morphology and potential ballooning-related changes. Ultrasound, magnetic resonance imaging (MRI), and transient elastography are valuable tools for clinicians to examine liver structural changes without requiring invasive procedures. These techniques can detect hepatocellular swelling, alterations in parenchymal echogenicity, and changes in liver stiffness that may accompany hepatocytic ballooning. Concurrently, the evolving landscape of biomarkers has opened promising avenues for understanding the dynamics of hepatocytic ballooning. Circulating microRNAs, small non-coding RNAs involved in post-transcriptional regulation, have demonstrated potential as biomarkers that reflect cellular stress and injury associated with ballooning. These microRNAs, detectable in blood samples, offer the advantage of minimally invasive monitoring. Additionally, lipidomic profiles have garnered attention due to the central role of lipid metabolism in NASH. Lipidomics can provide insights into the changes occurring within ballooned hepatocytes and their metabolic disturbances by analyzing the composition of lipids within biological samples [30].

Clinical Significance of Hepatocytic Ballooning in NASH Diagnosis and Prognosis

Hepatocytic ballooning is pivotal as a diagnostic and prognostic tool within the intricate landscape of NASH. Given that NASH exists along a spectrum of liver diseases within the NAFLD spectrum, accurate identification and assessment of its severity are crucial for effective clinical management [27].

Hepatocytic ballooning is a fundamental diagnostic criterion for distinguishing NASH from the milder form of simple steatosis. This distinction is paramount, as it guides clinicians toward appropriate treatment strategies. The presence and severity of hepatocytic ballooning provide a clear histological marker that assists in making this critical differentiation. Clinicians can tailor interventions involving lifestyle modifications, pharmacotherapy, or closer monitoring based on recognizing NASH's distinctive pathology [31].

The clinical significance of hepatocytic ballooning extends beyond diagnosis and encompasses disease prognosis. The extent of ballooning has emerged as a robust predictor of disease progression and adverse clinical outcomes. Research consistently demonstrates a correlation between the severity of ballooning and the advancement of fibrosis. As the stage of fibrosis is a critical determinant of long-term outcomes in NASH, the ability to assess ballooning offers valuable insights into the disease's trajectory. Moreover, ballooning has been linked to an increased risk of cirrhosis development and higher mortality rates. This knowledge enables clinicians to identify patients at greater risk and implement more vigilant monitoring and management strategies [32].

Understanding the clinical implications of hepatocytic ballooning also facilitates personalized medicine approaches. By evaluating the severity of ballooning and its association with disease progression, clinicians can tailor treatment strategies to individual patients. Those with more severe ballooning may require more aggressive interventions, while those with milder ballooning might benefit from less invasive approaches. This personalized approach optimizes the allocation of healthcare resources and enhances patient outcomes [33].

Association Between Hepatocytic Ballooning and Other Histological Features of NASH

Hepatocytic ballooning's significance extends beyond its standalone histological manifestation, as it demonstrates intricate connections with other defining hallmarks of NASH. The relationship between hepatocytic ballooning, inflammation, and fibrosis underscores its role in NASH pathogenesis [34].

In inflammation, ballooned hepatocytes play an active role in initiating and perpetuating the inflammatory cascade. These stressed cells release inflammatory cytokines, including interleukin-1β and tumor necrosis factor-α, which act as signaling molecules to attract and activate immune cells within the liver parenchyma. This immune cell infiltration further amplifies tissue injury and inflammation, contributing to the NASH phenotype. The interplay between ballooning and inflammation creates a self-reinforcing loop, as inflammation-induced stress exacerbates ballooning, and ballooned hepatocytes, in turn, perpetuate inflammation [35].

Moreover, the spatial relationship between ballooning and fibrosis is particularly intriguing. Hepatocytic ballooning is often observed near regions of fibrosis within liver tissue. This spatial association suggests a dynamic interplay between ballooning and fibrogenesis. While the exact nature of this relationship is still under investigation, it is plausible that the stress-induced cellular alterations associated with ballooning contribute to the fibrogenic response and vice versa. This close spatial arrangement also raises the possibility that hepatocytic ballooning might trigger fibrosis development in specific microenvironments [36].

Importantly, clinical studies have consistently demonstrated a correlation between the severity of hepatocytic ballooning and the degree of inflammation, fibrosis stage, and overall disease severity in NASH patients. The extent of hepatocytic ballooning indicates disease progression and is often used with other histological features to stratify NASH patients based on disease severity. This correlation highlights the intricate interplay between ballooning and the broader pathophysiological processes driving NASH evolution [37].

Current understanding of hepatocytic ballooning in NASH

Exploring Hepatocytic Ballooning in NASH Patients

The intricate role of hepatocytic ballooning in NASH has garnered significant attention from researchers worldwide. Numerous studies have attempted to decipher the clinical and molecular dimensions of hepatocytic ballooning in NASH patients. These studies, conducted across diverse demographics and populations, have collectively contributed to a more comprehensive understanding of this histological feature [38].

Histopathological analyses, at the core of these studies, have revealed the prevalence and clinical significance of hepatocytic ballooning in various cohorts of NASH patients. Researchers have meticulously examined liver tissue specimens from a wide spectrum of NASH cases, from early-stage to advanced disease. Utilizing advanced staining techniques and microscopic analyses, these studies have unveiled the distinct morphological alterations characterizing ballooned hepatocytes. This histopathological evidence confirms the presence of hepatocytic ballooning in NASH and underscores its role in distinguishing NASH from simpler forms of NAFLD [4].

Beyond the histopathological domain, these studies have delved into the molecular intricacies underlying hepatocytic ballooning. Researchers have gained insights into potential drivers of ballooning formation by analyzing gene expression profiles, signaling pathways, and cellular responses. These investigations have revealed associations between the severity of ballooning and the activation of pathways involved in oxidative stress, apoptosis, and immune responses. Furthermore, they have uncovered linkages between ballooning and the broader pathological cascades driving NASH progression, such as fibrogenesis and inflammation [39].

Perhaps the most compelling revelation from these studies lies in the direct correlation between the severity of hepatocytic ballooning and the trajectory of disease progression. In-depth analyses of ballooning severity and clinical outcomes have illuminated a direct relationship between more pronounced ballooning and adverse disease outcomes. These observations have fueled further exploration into the underlying mechanisms, with hepatocytic ballooning emerging as a potential predictor of NASH severity and progression [40].

Correlation Between Hepatocytic Ballooning and Disease Severity (Fibrosis Stage)

Accumulating evidence strongly supports a robust positive correlation between the presence and severity of hepatocytic ballooning and the overall disease severity in NASH. Extensive investigations across diverse patient cohorts consistently reveal that hepatocytic ballooning is closely associated with higher fibrosis stages. This observation suggests a compelling interplay between ballooning and fibrogenesis, potentially unveiling a critical mechanistic link between cellular stress and the architectural remodeling of the liver [41].

As NASH advances, the histological landscape within the liver transforms, with inflammation and fibrosis becoming increasingly prominent. Notably, hepatocytic ballooning tends to manifest concurrently with the escalation of fibrosis stages. This temporal association hints at a dynamic relationship between ballooning, fibrogenesis, and the orchestrated progression of NASH pathology. Interestingly, ballooning intensifies as fibrosis advances, possibly signifying an amplification loop in which cellular stress drives further tissue injury and remodeling [37].

The observed relationship between hepatocytic ballooning and fibrosis stages underscores the potential of ballooning as a surrogate marker for NASH progression. Its close alignment with disease severity positions ballooning as a histological feature that reflects the dynamic changes occurring at both the cellular and tissue levels. As ballooning is readily identifiable through histopathological analyses, its integration into clinical practice could provide a valuable tool for assessing disease advancement and guiding therapeutic strategies [42].

However, while this correlation between hepatocytic ballooning and fibrosis stage is compelling, the precise molecular mechanisms governing their interaction remain elusive. Further research is warranted to dissect the intricate signaling pathways connecting ballooning, inflammation, and fibrogenesis, ultimately providing a comprehensive understanding of their interrelationship. By unraveling these complexities, we can identify novel therapeutic avenues targeting the fundamental processes driving NASH progression [43].

Inflammatory Mediators and Cellular Pathways Linked to Hepatocytic Ballooning

Hepatocytic ballooning, a hallmark of NASH, is intricately linked to the inflammatory landscape within the liver microenvironment. Inflammation plays a central role in developing and progressing hepatocytic ballooning, with proinflammatory cytokines and immune cell responses converging to shape this distinctive histological feature. Among the pivotal mediators in this intricate process are IL-1β and TNF-α, potent proinflammatory cytokines known for their key roles in immune regulation. These cytokines initiate a cascade of events within hepatocytes, triggering cellular stress responses and the ballooning phenotype. Specifically, they can disrupt normal cellular processes, such as lipid metabolism and mitochondrial function, ultimately resulting in hepatocellular injury and ballooning. This close association between hepatocytic ballooning and the actions of IL-1β and TNF-α underscores the significant impact of inflammation on the pathogenesis of NASH. It highlights these cytokines as potential therapeutic targets for mitigating ballooning and its detrimental consequences. Understanding these inflammatory mediators and their interconnected pathways is crucial for unraveling the complexities of NASH and advancing targeted treatment strategies [44].

Proinflammatory Cytokines: IL-1β and TNF-α

IL-1β and TNF-α, potent proinflammatory cytokines, are pivotal in driving hepatocytic ballooning by initiating cellular stress and apoptosis pathways. These cytokines interact with their respective receptors on the surface of hepatocytes, setting off downstream signaling cascades that ultimately result in stress responses within these liver cells. The activation of these pathways frequently leads to significant alterations in cytoskeletal components, causing cytoplasmic swelling and the distinctive morphological changes characteristic of ballooning hepatocytes [45]. This molecular interplay underscores the central role of inflammation in developing hepatocytic ballooning. It highlights the importance of understanding these signaling pathways in the context of NASH pathogenesis [45].

Immune Cell Infiltration and ROS Release

In addition to cytokine-mediated signaling, immune cells that infiltrate the liver, particularly macrophages and neutrophils, play a substantial role in developing hepatocytic ballooning. These immune cells release proinflammatory mediators, exacerbating cellular stress and contributing to the ballooning phenotype. Moreover, they release ROS, which adds another layer of complexity to the process. ROS, generated as byproducts of oxidative stress, further damage cellular structures and amplify the ballooning process. This interplay between immune cells and ROS-driven oxidative stress creates a feedback loop, fostering a microenvironment within the liver that is conducive to the progression of hepatocytic ballooning [46]. This complex interaction highlights the multifaceted nature of hepatocytic ballooning in NASH and underscores the importance of considering both cytokine signaling and immune responses in understanding its pathogenesis.

Collective Impact on NASH Pathogenesis

The intricate interplay between inflammation and hepatocytic ballooning accentuates their profound impact on the pathogenesis of NASH. Ballooning hepatocytes, subjected to the relentless assault of inflammation and oxidative stress, become increasingly susceptible to apoptosis and subsequent inflammation. In a cyclical manner, this resulting inflammation perpetuates the ballooning phenotype, creating a vicious cycle that fuels the progression of the disease. This collective impact underscores the centrality of hepatocytic ballooning in the intricate web of NASH development, where inflammation and ballooning establish a reciprocal relationship, driving cellular injury and the relentless progression of the disease [47]. This complex interplay underscores the significance of understanding these processes for developing targeted therapeutic interventions in NASH.

Molecular Mechanisms Underlying Hepatocytic Ballooning Including ER Stress and Lipotoxicity

Recent advancements in molecular research have shed light on the intricate mechanisms orchestrating hepatocytic ballooning in the context of NASH. A central player in this complex process is ER stress, triggered by the excessive accumulation of lipids and metabolic disturbances within hepatocytes. The ER is a crucial cellular organelle responsible for protein synthesis, lipid metabolism, and calcium homeostasis. In the presence of lipid overload, the ER's capacity to manage the folding and processing of proteins becomes overwhelmed, leading to ER stress. This molecular disturbance initiates a series of signaling events collectively known as the unfolded protein response (UPR), aimed at restoring ER homeostasis. However, sustained and chronic ER stress triggers cellular stress responses associated with ballooning, contributing to the ballooning phenotype. The resulting disruption of ER function plays a role in the altered cellular morphology characteristic of ballooning [48].

Lipotoxicity, another significant contributor, arises from the influx of free fatty acids into hepatocytes. As these fatty acids accumulate within the cells, they initiate a cascade of events that ultimately lead to oxidative stress, mitochondrial dysfunction, and apoptotic pathways. Oxidative stress, stemming from an imbalance between the production of ROS and antioxidant defense, damages cellular components, further exacerbating ballooning-associated injury. Simultaneously, mitochondrial dysfunction disrupts energy production and promotes the generation of ROS, intensifying cellular stress. The culmination of these cascades drives ballooned hepatocytes toward apoptosis, contributing to the inflammatory environment characteristic of NASH [49].

In addition to ER stress and lipotoxicity, dysregulated lipid metabolism, compromised autophagy, and dysfunctional cytoskeletal components are emerging as intricate participants in the orchestration of ballooning. Altered lipid metabolism contributes to excessive fat accumulation, fueling the ballooning phenotype. Autophagy, a cellular process responsible for recycling damaged organelles and proteins, is disrupted in ballooned hepatocytes, amplifying the cellular stress response. Furthermore, the integrity of the cytoskeleton, essential for maintaining cell shape and structure, is compromised, further accentuating the ballooning phenotype [50].

Gaps in knowledge and controversies

Variability in the Assessment and Interpretation of Hepatocytic Ballooning

Hepatocytic ballooning, a hallmark of NASH, poses a significant challenge due to its subjective evaluation, leading to interobserver variability among pathologists. This subjectivity arises from the visual nature of assessing histological slides, where different experts can interpret subtle morphological changes differently. This variability can result in inconsistent diagnoses and hinder research reliability. Standardized criteria defining hepatocytic ballooning parameters and severity can significantly reduce interobserver variability, enhancing research reproducibility and clinical accuracy. Clear guidelines for assessment would facilitate meaningful comparisons between studies, allowing for more robust conclusions regarding the relationship between hepatocytic ballooning and disease progression [51-53].

Lack of Standardized Criteria for Grading Hepatocytic Ballooning

The absence of universally accepted grading criteria for hepatocytic ballooning severity has led to variability in how it is defined and scored in different studies. This inconsistency affects the reliability of clinical assessments and research findings. To address this challenge, uniform grading criteria for hepatocytic ballooning severity should be established through collaborative efforts involving pathologists, clinicians, researchers, and regulatory bodies. Consensus on clear definitions, scoring systems, and assessment parameters would enhance the accuracy of NASH diagnosis and research comparability, providing more robust insights into the role of hepatocytic ballooning in disease progression [54-56].

Uncertainty Regarding the Progression of Hepatocytic Ballooning to More Advanced NASH Stages

The role of hepatocytic ballooning in the progression of NASH to more advanced stages, such as fibrosis and cirrhosis, remains uncertain. Longitudinal studies and comprehensive molecular analyses are needed to investigate whether hepatocytic ballooning precedes or occurs concurrently with the development of fibrosis and cirrhosis. Longitudinal studies tracking patients can provide insights into the temporal relationship between ballooning and disease progression. Molecular analyses, including genomics, transcriptomics, and proteomics, can help unravel the molecular events associated with ballooning severity and its impact on subsequent disease stages, shedding light on its role in NASH progression [57,58].

Impact of Genetic and Environmental Factors on Hepatocytic Ballooning Variability

Genetic and environmental factors influence the varying severity of hepatocytic ballooning in NASH. Genetic predisposition, driven by gene variations related to lipid metabolism, inflammation, and cellular stress responses, can increase susceptibility to ballooning. Influenced by diet, lifestyle, and environmental exposures, epigenetic modifications further mediate how genetic information is expressed and can impact ballooning development. Gut microbiota composition also plays a critical role in influencing metabolic processes, including lipid metabolism and inflammation. Dysbiosis, or an imbalance in gut microbes, has been linked to NASH and hepatocytic ballooning. Environmental factors, such as diet, sedentary lifestyles, and exposure to toxins, can exacerbate ballooning susceptibility by promoting hepatic lipid accumulation, cellular stress, and inflammation [59-61].

Future directions and research avenues

Table 1 outlines key future research directions in the study of hepatocytic ballooning, a significant feature in NASH. These research avenues include developing non-invasive assessment methods, exploring molecular pathways and therapeutic targets, integrating omics data for a comprehensive understanding, establishing animal models and in vitro systems, and conducting longitudinal studies to decipher the temporal relationship between ballooning and fibrosis progression. These research areas promise to advance our knowledge of NASH and improve clinical management strategies.

**Table 1:** Future directions and research avenues in the field of hepatocytic ballooning

Research Area	Description
Advancements in Non-Invasive Assessment of Hepatocytic Ballooning	Development of reliable, non-invasive methods, such as quantitative MRI techniques and elastography, to assess hepatocytic ballooning. These tools can provide insights into disease progression and treatment response without invasive procedures [62].
Unexplored Molecular Pathways and Potential Therapeutic Targets Related to Ballooning	Deeper exploration of the molecular pathways involved in hepatocytic ballooning to identify novel therapeutic targets. Investigating specific signalling cascades like the unfolded protein response and autophagy may lead to druggable targets for intervention, potentially altering NASH progression [63].
Integration of Omics Data (Genomics, Transcriptomics, Proteomics) to Understand Ballooning Heterogeneity	Comprehensive integration of multi-omics data (genomic, transcriptomic, proteomic) to understand the heterogeneity observed in hepatocytic ballooning. Molecular signatures associated with ballooning severity and progression may facilitate the discovery of predictive biomarkers and new pathways driving ballooning [64].
Animal Models and In Vitro Systems for Studying Hepatocytic Ballooning Dynamics	Development of accurate animal models and in vitro systems to mimic hepatocytic ballooning. Animal models can provide insights into the temporal sequence of ballooning and its impact on disease progression. In vitro platforms, like 3D cell cultures and organoids, offer controlled environments for studying cellular responses associated with ballooning [65].
Longitudinal Studies to Elucidate the Temporal Relationship Between Ballooning and Fibrosis Progression	Conducting longitudinal studies to capture the dynamic interplay between ballooning and fibrosis progression. Tracking the evolution of ballooning severity alongside the fibrosis stage over time can provide insights into whether ballooning is a precursor to fibrosis or a concurrent phenomenon, reshaping our understanding of NASH pathogenesis [66].

Therapeutic implications and clinical management

The recognition of hepatocytic ballooning's central role in the pathogenesis of NASH has significant therapeutic implications. Hepatocytic ballooning is a critical nexus where multiple key pathological processes converge, making it an attractive target for novel treatment strategies. By directing therapeutic efforts toward hepatocytic ballooning, the potential to disrupt the vicious cycle of NASH progression becomes evident [67].

One potential avenue for intervention is the alleviation of cellular stress. Hepatocytic ballooning reflects a state of heightened cellular stress, often resulting from factors such as lipotoxicity and ER stress. Targeting these stress pathways could attenuate ballooning-associated damage, thereby reducing the release of proinflammatory signals that exacerbate disease progression [68].

Enhancing autophagy, the cellular process responsible for degrading and recycling damaged cellular components offers another promising approach. Ballooned hepatocytes often exhibit impaired autophagy, which can perpetuate cellular dysfunction and inflammation. Therapeutic strategies that boost autophagic flux could restore cellular homeostasis and dampen ballooning-related inflammation [69].

Modulating inflammation associated with hepatocytic ballooning represents an additional therapeutic avenue. Inflammation is both a consequence and driver of ballooning, creating a self-perpetuating loop that fuels disease advancement. Interventions that mitigate the inflammatory response could attenuate ballooning's impact on hepatocyte injury, interrupting the cascade of events leading to fibrosis, cirrhosis, and their associated complications [70].

Current Therapeutic Strategies and Their Effects on Hepatocytic Ballooning

Current therapeutic strategies for NASH primarily focus on mitigating metabolic derangements and reducing the inflammatory milieu, which can indirectly impact hepatocytic ballooning. Lifestyle modifications encompassing dietary changes and increased physical activity aim to improve insulin sensitivity, reduce hepatic fat accumulation, and alleviate ballooning-related cellular stress. Weight loss, achieved through various interventions, reduces hepatocyte lipid content and ameliorates systemic inflammation, potentially attenuating ballooning severity [71].

Insulin sensitizers, including thiazolidinediones and GLP-1 receptor agonists, target insulin resistance, a key contributor to ballooning-associated lipotoxicity. These agents promote glucose uptake, improve lipid metabolism, and modulate inflammatory pathways, potentially curbing ballooning-related stress responses. Additionally, novel experimental treatments are emerging that directly address ballooning-associated pathways. Antifibrotic agents, such as FXR agonists and LOXL2 inhibitors, aim to disrupt the vicious cycle of inflammation and fibrosis, which could indirectly impact ballooning severity by altering the microenvironment of ballooned hepatocytes [72].

Furthermore, promising insulin sensitizers under investigation exhibit the potential to mitigate ballooning-associated pathways directly. These novel agents may intervene in the molecular cascades triggered by ER stress and oxidative stress, contributing to ballooning and cellular injury. Investigating the effects of these treatments on hepatocytic ballooning holds the potential to unravel their direct impact on cellular stress responses and provide insights into their efficacy in halting NASH progression [73].

Challenges in Developing Treatments Specifically Targeting Hepatocytic Ballooning

Developing treatments that directly target hepatocytic ballooning presents a formidable challenge due to the intricate and multifaceted nature of its underlying mechanisms. Hepatocytic ballooning is not an isolated phenomenon but emerges downstream from a cascade of complex cellular processes. These include ER stress, lipotoxicity, and inflammation, each contributing to the cellular stress and dysfunction characterizing ballooning [74].

The challenge lies in designing therapeutic agents that can effectively intervene at multiple levels of these interconnected pathways. ER stress, for instance, triggers an UPR that can exacerbate ballooning-associated cellular stress. Lipotoxicity, driven by the accumulation of excessive lipids within hepatocytes, further amplifies oxidative stress and inflammation, contributing to ballooning. As such, any therapeutic strategy targeting ballooning must encompass these diverse factors, necessitating a comprehensive understanding of their intricate interplay [75].

Moreover, the intricate network of molecular interactions within these pathways requires a nuanced approach. An intervention aimed at one aspect of ballooning-related mechanisms might inadvertently impact others. This underscores the importance of precision in therapeutic targeting to avoid unintended consequences and optimize treatment outcomes [76].

Furthermore, the complexity of the liver's physiology and its role in various metabolic processes adds another layer of complexity. The potential for off-target effects and unintended consequences underscores the need for a deep mechanistic understanding to guide therapeutic development. Preclinical models that accurately recapitulate the ballooning phenotype and its associated mechanisms are crucial for effectively evaluating potential therapies [77-80].

## Conclusions

In conclusion, this comprehensive review underscores the paramount significance of hepatocytic ballooning within the intricate landscape of NASH. Our exploration has traversed the realms of its histological manifestations, clinical ramifications, and the intricate web of underlying molecular pathways. It is abundantly clear that hepatocytic ballooning is not a mere bystander but an orchestrator of pivotal events in the progression of NASH. This histological feature's interconnectedness with inflammation, fibrosis, and the overall advancement of the disease has been unequivocally illuminated, solidifying its pivotal role. As we navigate the complex terrain of hepatocytic ballooning, it becomes evident that our journey to fully unravel its intricacies is ongoing. The identified knowledge gaps, ranging from variations in assessment methodologies to the absence of standardized criteria, serve as beacons guiding us toward further exploration. In this quest, collaboration emerges as the cornerstone of progress. The convergence of efforts among researchers, clinicians, and industry stakeholders is not just beneficial; it is imperative. Together, we can decipher the enigmatic mechanisms at play, forge innovative diagnostic tools, and pinpoint precise therapeutic targets. Through these collaborative endeavors, we hold the potential to expedite the translation of our findings into tangible advancements for the management of NASH. In essence, hepatocytic ballooning stands as a vital piece in the intricate puzzle of NASH. It is through the collective efforts of the scientific community that we can piece together this puzzle, charting a course toward elevated patient care and a more comprehensive understanding of NASH. The path ahead may be challenging, but with dedication and collaboration, we are poised to make meaningful strides in the fight against NASH.
